# Identifying Candidate Genes for Litter Size and Three Morphological Traits in Youzhou Dark Goats Based on Genome-Wide SNP Markers

**DOI:** 10.3390/genes14061183

**Published:** 2023-05-29

**Authors:** Xiaoyan Sun, Qunhao Niu, Jing Jiang, Gaofu Wang, Peng Zhou, Jie Li, Cancan Chen, Liangjia Liu, Lingyang Xu, Hangxing Ren

**Affiliations:** 1 Chongqing Academy of Animal Sciences, Rongchang 402460, China; sddun@foxmail.com (X.S.); jiangjing19@163.com (J.J.); wanggaofs20031216@163.com (G.W.); cqzp2006@163.com (P.Z.); cqhylijie@163.com (J.L.); 18712378145@163.com (C.C.); liulj619@163.com (L.L.); 2 Institute of Animal Sciences, Chinese Academy of Agricultural Sciences, Beijing 100193, China; nqh_5195@163.com

**Keywords:** litter size, morphological traits, genome-wide association study, selection signature analysis, runs of homozygosity, Youzhou dark goat

## Abstract

This study aimed to reveal the potential genetic basis for litter size, coat colour, black middorsal stripe and skin colour by combining genome-wide association analysis (GWAS) and selection signature analysis and ROH detection within the Youzhou dark (YZD) goat population (n = 206) using the Illumina GoatSNP54 BeadChip. In the GWAS, we identified one SNP (snp54094-scaffold824-899720) on chromosome 11 for litter size, two SNPs on chromosome 26 (snp11508-scaffold142-1990450, *SORCS3*) and chromosome 12 (snp55048-scaffold842-324525, *LOC102187779*) for coat colour and one SNP on chromosome 18 (snp56013-scaffold873-22716, *TCF25*) for the black middorsal stripe. In contrast, no SNPs were identified for skin colour. In selection signature analysis, 295 significant iHS genomic regions with a mean |iHS| score > 2.66, containing selection signatures encompassing 232 candidate genes were detected. In particular, 43 GO terms and one KEGG pathway were significantly enriched in the selected genes, which may contribute to the excellent environmental adaptability and characteristic trait formation during the domestication of YZD goats. In ROH detection, we identified 4446 ROH segments and 282 consensus ROH regions, among which nine common genes overlapped with those detected using the iHS method. Some known candidate genes for economic traits such as reproduction (*TSHR*, *ANGPT4*, *CENPF*, *PIBF1*, *DACH1*, *DIS3*, *CHST1*, *COL4A1*, *PRKD1* and *DNMT3B*) and development and growth (*TNPO2*, *IFT80*, *UCP2*, *UCP3*, *GHRHR*, *SIM1*, *CCM2L*, *CTNNA3* and *CTNNA1*) were revealed by iHS and ROH detection. Overall, this study is limited by the small population size, which affects the results of GWAS to a certain extent. Nevertheless, our findings could provide the first overview of the genetic mechanism underlying these important traits and provide novel insights into the future conservation and utilisation of Chinese goat germplasm resources.

## 1. Introduction

Litter size is a crucial index for estimating female goats’ reproductive performance and has aroused increasing interest from husbandry producers and breeders. Simultaneously, morphological characteristics have also increasingly attracted attention because they can be correlated with other economically relevant traits in goats and sheep. During the past decade, various efforts have been made to identify the genetic variations in litter size [1,2], coat colour, skin colour [3,4] and other economic traits in different goat or sheep breeds. However, the genetic basis of the same trait showed significant differences among or within different breeds, which is likely due to the complexity of the goat breed formation process and the polygenetic effects.

The YZD goat, one of the most famous Chinese indigenous goat breeds, has been primarily distributed in a mountainous and transportation-restricted region at an altitude of 800–1000 m in Youyang County, China, since approximately a hundred years ago. The long period of isolation in the environment shaped its distinctive character. The YZD goat has a small body size, with the adult male is merely 32–37 kg. Meanwhile, with the permeation of large meat goat breeds, raisers carried out excessive and disorderly crossbreeding, which reduced the YZD goat population and endangered it. In 2009, it was certified as a national livestock genetic resource. It is characterised by unique exterior pigmentation traits (the whole body’s dark skin and dark visible mucous membranes and a white coat with a black middorsal stripe [5]) (Supplementary File S1). After long-term natural and artificial selection, the YZD goats exhibit excellent adaptability to the humid environment of the mountain area in Chongqing Province and remarkably contribute to local livestock husbandry. Our team previously investigated the meat quality and other characteristics of YZD goats and revealed the nutritional basis of their excellent meat flavour [6], thus laying the foundation for developing it into high-grade goat meat in the market. The genetic diversity of native goats is essential to promote the conservation and sustainable development of germplasm resources. Nevertheless, there is a lack of investigation on genome-wide population genetic structure analysis and characteristic trait exploration for YZD goats. As such, the underlying molecular mechanisms of the peculiar YZD goat phenotype remain elusive and warrant further in-depth study.

In the present study, we explored the putative candidate genes and SNPs related to litter size and three morphological traits (coat colour, black middorsal stripe and skin colour) within the YZD goat population by combining a GWAS, selection signature analysis (iHS) and runs of homozygosity detection (ROH). Our study could provide novel insights into the molecular mechanisms underpinning these traits in Chinese native goat breeds, thereby contributing to their further utilisation.

## 2. Materials and Methods

### 2.1. Sampling and Genotyping Quality Control

Blood samples were collected from 206 adult goats from the YZD goat breed conservation farm in Chongqing Province, Southwest China. For the litter size trait, only the data from female goats (n = 154) aged 1 to 4 years were analysed in the GWAS. Each trait was categorised as one or zero. The litter size trait was classified as one to two lambs (n = 147) or more than two lambs (n = 7) based on the lambing record. We divided the three morphological traits according to presence or absence and included pictures for better understanding (Supplementary File S1). The trait classification and sample number are shown in Table 1 and the detailed phenotype data are also presented in Table S1. Genomic DNA was extracted and purified from whole blood using a QIAamp DNA blood mini kit (Qiagen). According to the manufacturer’s instructions, the qualified DNA was genotyped using the Illumina GoatSNP50 BeadChip (Illumina Inc., San Diego, CA, USA) containing 53,346 SNPs [7]. The genotypic data are shown in Table S2. After genotyping, quality control was implemented using PLINK 1.07 [8]. The quality control condition of SNP markers was set as (i) sample call rate > 90%, (ii) SNP call rate > 90%, (iii) a minor allele frequency (MAF) of >5% per SNP and (iv) Hardy–Weinberg equilibrium (HWE) > 0.000001. Following these quality control procedures, 205 individuals and 41,180 SNPs were retained for further GWAS and selection signature analysis.

### 2.2. Genome-Wide Association Studies

We performed genome-wide association studies using the linear model (LM) implemented in Plink 1.07 [8]. The corrected significant *p* values were used for the following association analyses. The population structure was investigated using ADMIXTURE 1.3 [9]. The ggplot2 in the R package generated quantile-quantile (Q-Q) plots and Manhattan plots from the GWAS results. The genome-wide association significance threshold was *p* < 1.21 × 10^−6^ (0.05/41,180). Based on the *Capra hircus* reference genome GCA_000317765.1 (CHIR 1.0) in genome data viewer from the National Center for Biotechnology Information (NCBI), putative candidate gene(s) were identified within approximately 100 kb upstream and downstream of the most significant SNPs.

### 2.3. Selection Signature Detection Using the iHS Method

We employed the iHS method to detect strong footprints of recent positive selection within the YZD goat population. Beagle v3.3.2 (Auckland, Zealand) [10] was used to impute missing alleles and infer the haplotype phase according to the SNPs obtained after quality control. The normally standardised iHS was calculated as

$$iHS=\frac{\ln\left(\frac{iHH_{A}}{iHH_{D}}\right)-E_{p}[ln(\frac{iHH_{A}}{iHH_{D}})]}{SD_{p}[ln(\frac{iHH_{A}}{iHH_{D}})]}$$

where $iHH_{A}$ and $iHH_{D}$ represent the integrated EHH score for ancestral and derived core alleles, respectively. $E_{p}[ln(\frac{iHH_{A}}{iHH_{D}})]$ and $SD_{p}[ln(\frac{iHH_{A}}{iHH_{D}})]$ are the expectation and the standard deviation in frequency bin *p*, respectively. Single-site iHS values were computed across the genome in the goat population. Then, the norm module of Selscan was applied to normalise the iHS score. SNPs with |iHS| scores higher than 2.66 (the top 1%) were considered to have candidate selection signatures. The windows 50 kb upstream and downstream of candidate signatures were considered the selected regions to capture the putative genes. We then analysed the biological functions of the selected genes by Gene Ontology (GO) annotation and Kyoto Encyclopedia of Genes and Genomes (KEGG) pathway enrichment using the online tool DAVID 2021 (https://david.ncifcrf.gov/tools.jsp) (accessed on 1 December 2021). Human gene ontologies were used in this study, as the goat genome was not adequately annotated. In addition, the human genome is more annotated than other species, thus improving the probability of retrieving GO terms for the goat genome. Afterwards, we conducted a comprehensive review of the literature to annotate the functions of the identified genes.

### 2.4. ROH Detection

We used PLINK v1.90 [8] to detect ROHs for each individual and estimated the consensus regions to explore the potential genes related to traits under investigation. Several important parameters for defining ROHs were as follows: (i) the minimum length was 500 kb; (ii) the proportion of homozygous overlapping windows was 0.05; (iii) the minimum number of consecutive SNPs included in an ROH was 20; (iv) the minimum SNP density was 100 kb/SNP; (v) the maximum gap between continuous homozygous SNPs was 100 kb; and (vi) a maximum of two SNPs with missing genotypes and up to one heterozygous genotype were allowed in an ROH. We then annotated genes within the consensus ROHs and compared them to the iHS results to identify the overlapping genes.

## 3. Results

### 3.1. Genome-Wide Association Studies and Annotation of Candidate Genes

**Figure 1:**
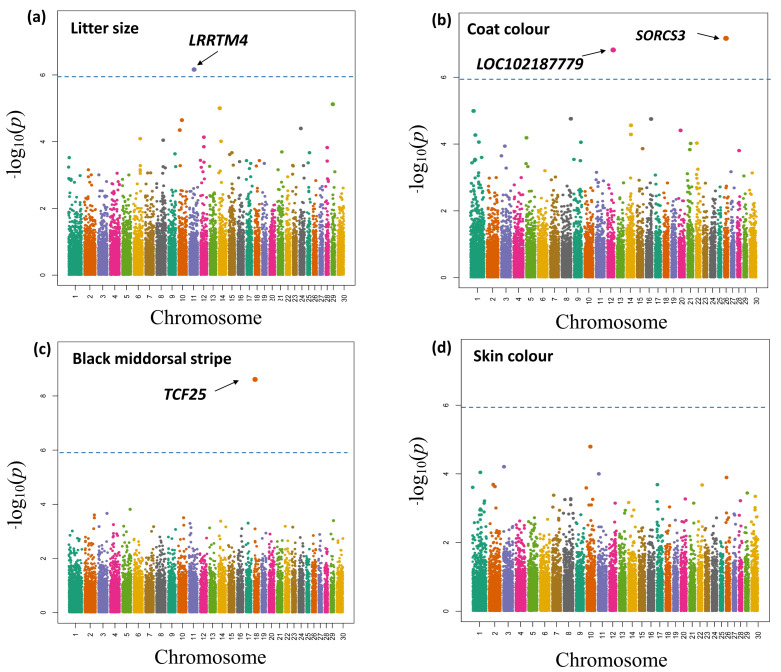
Manhattan plots showing the genome-wide distribution of SNPs for four traits in the YZD goat breed. (**a**) Litter size; (**b**) Coat colour; (**c**) Black middorsal stripe; (**d**) Skin colour. The blue horizontal lines indicate the thresholds at the genome-wide level.

The PCA indicated no apparent family differentiation in the selected goats, which was suitable for subsequent GWASs (Figure S1). The Q-Q plots displayed a reasonable *p*-value distribution (Figure S2), suggesting no genomic inflation or systematic bias in this study. We then annotated all SNPs significantly associated with each trait at the genome-wide level (Tables S3–S6). The GWAS results were visualised by Manhattan plots (Figure 1) and are summarised in Table 2. In detail, this included one significant SNP on chromosome 11 (150 kb from *LRRTM4*) for litter size (Figure 1a), one significant SNP on chromosome 26 (*SORCS3*) and one significant SNP on chromosome 12 (*LOC102187779*) for coat colour (white vs. brown) (Figure 1b) and one significant SNP on chromosome 18 (*TCF25*) associated with the black middorsal stripe (Figure 1c). In contrast, no SNPs were identified for skin colour (Figure 1d).

### 3.2. Selection Signatures Detected by iHS

**Figure 2:**
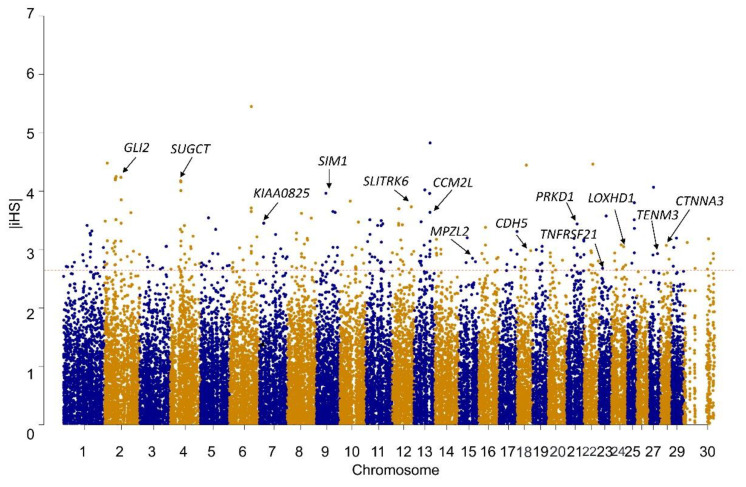
Genome-wide distribution of selection signatures detected by iHS analysis in YZD goats. The horizontal line indicates the threshold level of the top 1% (|iHS| = 2.66) and alternating colours distinguish markers on neighbouring chromosomes.

**Figure 3:**
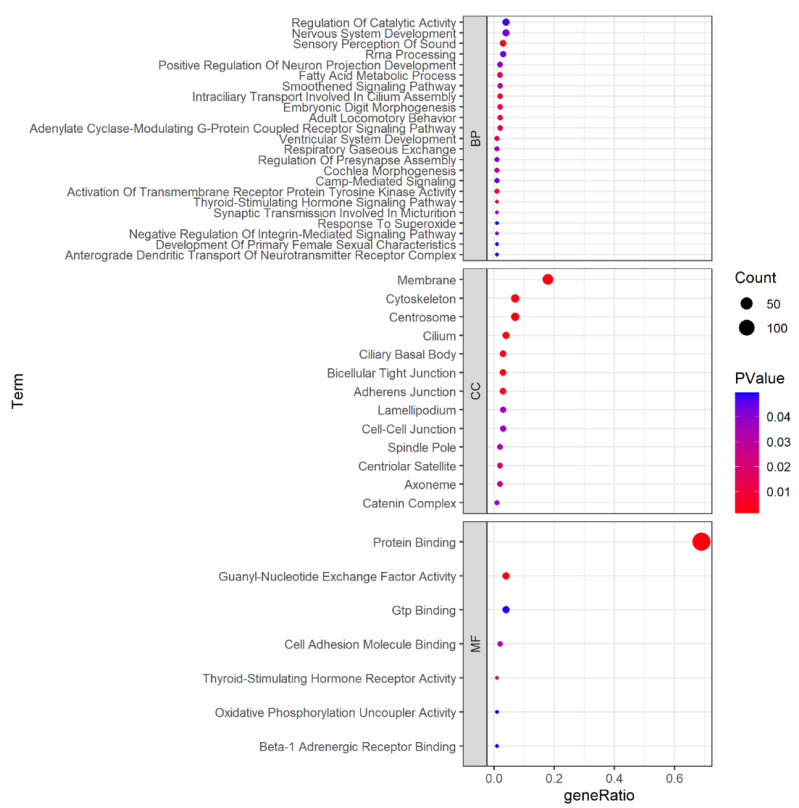
Gene ontology (GO) categories of the selected genes detected in YZD goats by iHS.

In total, 29,459 SNPs were obtained with estimated |iHS| scores (Table S7). The scans of |iHS| for the YZD goat population are shown in Figure 2. The plots show clear evidence of selective forces acting in different genome regions. The region located on CHR6 (82,856,006–82,956,006) had the highest |iHS| score (5.45) and no candidate gene overlapped with this region. A total of 295 candidate regions meeting the threshold of the top 1% were identified, among which 232 candidate genes were annotated (Table S8) and used for subsequent functional enrichment analysis. There were 43 significant GO terms (22 biological processes, 7 molecular functions and 13 cellular components) and one significant KEGG pathway (*p* < 0.05), as visualised in Figure 3 and summarised in Table S9. The top 20 significant biological processes GO items demonstrate some biological functions, including the sensory perception of sound, activation of transmembrane receptor protein tyrosine kinase activity, fatty acid metabolic process, nervous system development, regulation of catalytic activity and others. Notably, some selected genes were previously reported to be associated with economically important traits and are discussed below. Furthermore, we marked several genes with the highest iHS scores in Figure 2 and searched the literature to explore their function.

### 3.3. ROH Results

#### 3.3.1. Genomic ROH Distribution

**Figure 4:**
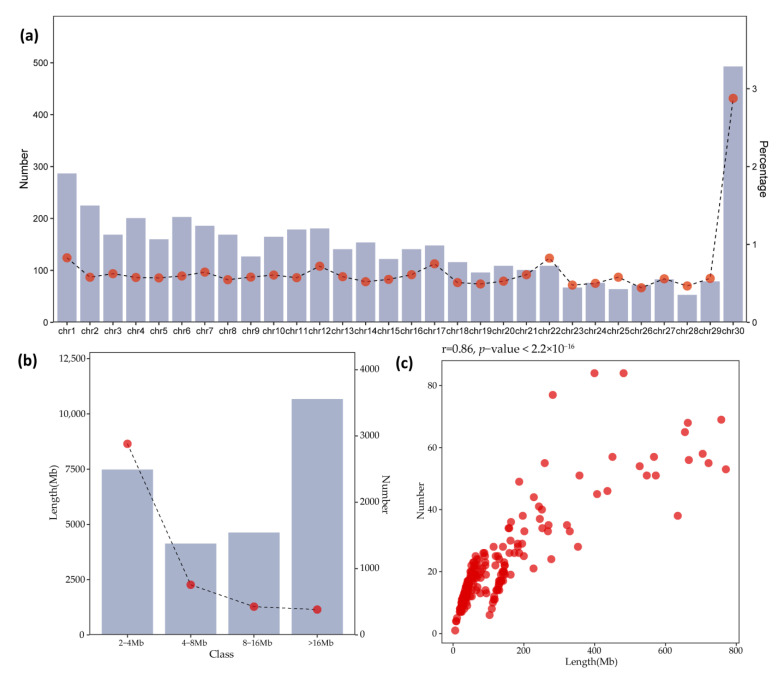
(**a**) Number of ROHs per chromosome (bars) and average percentage of each chromosome covered by ROHs (orange line); (**b**) The total numbers and lengths of runs of homozygosity (ROHs) of three classes; (**c**) Correlation of total ROH numbers and lengths. The ROH number (y-axis) is plotted against the ROH length (x-axis, i.e., length in Mb covered by ROH) in each individual.

We identified a total of 4446 ROH segments from 205 YZD goats (Table S10). For each animal, the average number of ROHs was 21.68, with an average length of 13.10 MB, covering a relatively small portion of the genome. The average length of ROHs was 5.10 Mb across all chromosomes, but the total length of ROHs per individual varied from 6.04 Mb to 77.10 Mb. (Figure 4). The longest segment was found on CHR1 (79.19 Mb with 1341 SNPs), while the shortest ROH was detected on CHR2 (2.0 Mb with 27 SNPs). The percentage of goat chromosomes covered by ROHs is shown in Figure 4a. The highest coverage by ROHs was observed on CHR30 (4.31% of the chromosomal length), whereas the lowest coverage was on CHR28 (0.66% of the chromosomal length). The number of ROHs per chromosome was greatest for CHR30 (492 segments) and lowest for CHR28 (52 segments).

#### 3.3.2. Genomic Patterns of ROHs

To evaluate the genomic pattern of ROH, we classified ROH into four categories according to their physical length: 2 to 4 Mb, 4 to 8 Mb, 8 to 16 Mb and >16 Mb (Figure 4b). Descriptive statistics of each length category are given in Table 3. Our results show that the total length of ROH for the YZD goat population was composed mostly of shorter segments (2 to 4 Mb), with a total length of 7466.24.37 Mb and these segments accounted for approximately 64.82% of all ROH detected. Moreover, we found that the total lengths of ROH and the numbers of ROH were highly correlated (r = 0.86, *p* < 2.2 × 10^−16^) (Figure 4c).

#### 3.3.3. The Consensus ROH Regions and the Genes Overlaping with Those Detected on the Basis of iHS

We found 282 consensus ROH regions and annotated 925 genes. It is worth noting that there were nine common genes revealed by both iHS and ROH analyses: *REM1*, *MAGI2*, *DNMT3B*, *ACSF3*, *FGGY*, *CTNNA1*, *P4HA2*, *GRM7* and *CHL1*. The majority of the nine common genes were related to reproduction, fat deposition, growth and development, meat quality, milk flavour and environmental adaptation.

## 4. Discussion

### 4.1. Litter Size

Litter size is one of the most critical reproductive traits in goats and sheep, showing variation among breeds and remaining incompletely understood. Initially, no gene was found within the regions 100 kb upstream and downstream of the significant SNPs in this study. We then extended the region to 150 kb and found that the nearest gene was *LRRTM4*. *LRRTM4* is essential for excitatory synaptic transmission and was proven to be associated with human mental disease. Genome-wide analysis in Brazilian cattle breeds showed that this gene is involved in biological processes related to fertility [11]. The selection signature analysis and ROHs also revealed some genes previously reported to be associated with litter size and other female animal reproduction-related traits, e.g., *TSHR* (seasonality of reproduction in modern domesticated goats) [12], *ANGPT4* (bovine ovarian antral follicle development) [13], *CENPF* (early embryonic development in mice and bovines) [14,15], *CHST1* (goat and sheep prolificacy) [16], *PIBF1* (embryos quality and development) [17], *DACH1* and *DIS3* (sheep litter size) [18], *COL4A1* (sheep prolificacy) [19], *PRKD1* (pig litter size) [20] and *DNMT3B* (first-born litter size in goats) [21].

Several candidate genes related to litter size or reproduction traits have been identified using data from resequencing or SNP chips. Numerous GWASs studies have reported some genetic markers associated with goat litter size and prolificacy, such as *GDF9*, *BMP15* and *BMPR1B*. However, large-effect markers for litter size that can be used in a wide range, such as *FecB* in most sheep breeds, have not been found in goats thus far. This may be due to the low heritability of litter size traits in most domestic animals, genetic complexity and nutritional and environmental factors.

### 4.2. Coat Colour

Coat colour is an economically important trait in domesticated goat breeds, particularly for wool/hair production. In fact, domestic goats are characterised by high variability of coat colour phenotypes due to human selection, so identifying the genetic loci affecting coat colour in goats has long been of interest. In YZD goats, most individuals generally possess white coat hair, while some have brown coat hair. The genetic mechanism underlying this coat colour segregation has not yet been fully elucidated.

The *SORCS3* gene was proven to be related to the pathophysiology of destructive diseases of the nervous system, leading to the occurrence and development of brain diseases [22]. Studies have shown that the *SORCS3* gene is associated with temperament traits, energy balance, feed efficiency and backfat thickness in cattle, mice, broilers and Duroc pigs, respectively [23,24,25,26]. Since melanocytes and the nervous system share a common developmental origin, we presume that the *SORCS3* gene may be associated with regulating melanocyte function, thereby participating in the coat colour determination in YZD goats. It is well established that coat colour variation is likely determined by the biochemical function, availability and distribution of eumelanin (black/brown) and pheomelanin (yellow/red) in mammals [27], which are commonly controlled (regulated) by the *ASIP* and *MC1R* genes [4]. In addition to *MC1R* and *ASIP*, other well-known genes have been widely confirmed to be associated with coat colour variation in various goat and sheep populations, such as *TYRP1*, *MITF*, *KIT*, *RALY*, *KITLG* and *GLI3* [28,29,30]. In short, the genetic loci that underlie different coat colour phenotypes are still elusive in goats. However, the mechanisms by which the *SORCS3* gene affects coat pigmentation in YZD goats remain to be addressed. In addition, the biological importance of uncharacterised *LOC102187779* remains to be elucidated.

### 4.3. Black Middorsal Stripe

In addition to YZD goats, the black middorsal stripe phenotype can also be found in other domesticated and wild animals, such as Chinese hamsters, sugar gliders and slow lorises. Therefore, it is indispensable to elucidate the genetic basis of this special coat pattern.

T-cell transcription factors (TCFs) are essential regulators of Wnt-induced transcriptional activation. Interestingly, Wnt is an important upstream signal in melanogenesis [31]. This finding suggests that TCF is likely involved in the modulation of pigmentation. Indeed, several investigations have demonstrated that TCF participates in hair pigmentation [32] or melanocyte biology [33]. In mouse embryos, TCF25 is intensely expressed in the dorsal root ganglia [34]. In addition, our ROH result revealed a genomic region (CHR 18: 14,190,132 to 14,531,916 bp) containing the *TCF25* and *MC1R* genes. Given this, we speculated that the regional differential expression of TCF25 might affect the subsequent melanocyte migration, leading to the black middorsal stripe in goats. Our results provide novel insight into the genetic factors underlying black middorsal stripe traits in animals. Nevertheless, the association between the black middorsal stripe and the *TCF25* gene remains to be addressed further.

### 4.4. Skin Colour

A previous study in mice demonstrated that changes in skin colour depend on different processes that occur after neural crest differentiation but before mature hair growth [35]. Additionally, it was demonstrated that human skin pigmentation is complex, the genetic architecture varies by latitude and known pigmentation loci explain only a small fraction of the variance [36]. Likewise, similar outcomes have been reported in investigations in sheep and cattle [37,38]. Although some progress has been made in the elucidation of the genetic basis of skin pigmentation in humans [39], chickens [40,41], cattle [42], sheep [43] and so on, the genetics of skin colour are currently less understood than those of hair colour.

To our surprise, we did not detect any SNPs for skin colour by the GWAS approach in this study, given the apparent differences in the phenotype of skin colour. Some SNP markers specific to some local goat breeds could have been absent from the commercial chip (Illumina GoatSNP50 BeadChip) when the manufacturer designed it. However, our team recently identified a presumed structural variation (duplication) in the *ASIP* gene by de novo sequencing of the YZD goat genome, which might be responsible for its lower expression in the hyperpigmented skin of YZD goats. Further investigations are ongoing in our laboratory to reveal the real genetic variation of dark skin colour in YZD goats.

### 4.5. Selection Signatures in YZD Goats

Detecting recent positive selection signatures in domesticated animals that have undergone both artificial and natural selection can contribute to the identification of beneficial mutations for economically important traits. In this study, we found that some GO terms can well mirrored the journey of domestication or indicated the formation of pigment phenotypes. For instance, the adenylate cyclase-modulating G-protein coupled receptor signalling pathway (GO:0007171) and cAMP-mediated signalling (GO:0019933) both participate in the regulation of melanogenesis and melanocyte biology, which may contribute to the pigmentation of hair and skin in YZD goats.

Furthermore, the genes enriched in GO terms and genes corresponding to the highest iHS scores were reported to be associated with various properties, including visual impairment (*SLITRK6*, *TNFRSF21*, *TENM3* and *FAM161A*,) [44,45,46,47], hearing loss (*SLITRK6*, *SLC52A3*, *SLC26A4*, *MPZL2*, *LOXHD1 and GRM7*) [48,49,50,51,52] and neural development (*TSHR*, *C2CD3* and *NRG1*) [30], which possibly reflects the most critical changes in the brain and neural development traits that occurred during the early stage of domestication in YZD goats. Sight, hearing and neural development are essential for goat survival in the wild. Some traits, such as reduced brain size, impaired vision and hearing and blunted sensory systems, were selected and fixed, making goats easy to capture and domesticate.

In addition to the genes related to early domestication, a panel of interesting genes associated with other economic traits was also revealed. These genes were previously reported in goats, sheep and other species, showing correlations with spermatogenesis and sperm motility (*KIF3B*, *KIF17*, *KIAA0825* and *TNFRSF21*) [53,54,55,56], growth and development (*TNPO2*, *IFT80*, *UCP2*, *UCP3*, *GHRHR*, *SIM1*, *CCM2L*, *CTNNA3* and *CTNNA1*) [57,58,59,60,61,62,63,64], fat deposition (*ACSF3* and *FGGY*) [65,66], chicken heat tolerance (*TSHR* and *SUGCT*) [67], high-altitude hypoxia adaptation (*CHL1*) [68], milk composition (*GHRHR*) [69], milk flavour (*ACSF3*) [70], carcass traits (*GHRHR*) [71] and hair follicle morphogenesis (*GLI2*) [72]. The iHS and ROH methods both detected some genes, but each method found additional genes not detected by the other. This confirms the necessity of using several tools to achieve more comprehensive and reliable results, as different signals were more easily detected by one of the tools. Therefore, the captured genes revealed by selection signature analysis implied that the reproductive, growth and development traits of YZD goats were affected by the human selection process and provided clues for subsequent studies on other economic traits in YZD goats.

This study detected some genomic loci and presumed genes related to agricultural economic characteristics by combining genomic approaches and could provide a reference for further conservation and utilisation of YZD goat germplasm resources and the knowledge of candidate genes can be incorporated into future genome selection strategies. For instance, the YZD goat is a kind of natural skin colour mutant that is rare in other mammals. Therefore, genetic analysis of its unique black skin could potentially provide a medical model to study human melanin-related diseases, such as skin melanopathy and mucosal melanosis. In addition, some consumers prefer dark-haired and/or dark-skinned livestock and poultry, such as black goats and black-bone silky chicken. Research on goat skin colour can help improve its competitiveness in the goat meat market. Moreover, the current small population limits the application of the genomic selection strategy, so identifying litter size-related candidate regions/genes may provide an opportunity to accelerate population expansion.

Of course, this study did have some limitations. First, the GWAS was based on relatively small sample size, a common flaw in numerous previous studies on native goat breeds [73,74]. The small sample size hindered the detection of robust and reliable association signals and only suggested that identified significant SNPs and/or mapped genes may be associated with the studied traits, which undoubtedly requires further verification in a larger YZD goat population. Second, insufficient SNP coverage could lead to low detection efficiency. Using whole-genome resequencing to obtain high-density markers is essential in future research on economic trait mechanisms in domestic animals. Third, due to the unbalanced trait classification, the standard GWAS software and simple linear model may not handle the association analyses well. Therefore, complex models that consider more covariates and software better at dealing with unbalanced binary traits, e.g., ASReml and SAIGE, should be applied to optimise the positioning accuracy and reliability of GWASs. Finally, combining whole genome re-sequencing and multi-omics analysis in large populations is indispensable for accelerating future genomic breeding progress.

## 5. Conclusions

In conclusion, this study preliminarily identified some significant SNPs and/or putative candidate genes related to litter size and three morphological traits in YZD goats using SNP chip genotyping data. The results provide valuable references for understanding the genetic basis of these traits and will facilitate further potential conservation and utilisation in YZD goat production. However, further research is needed to fully understand the genetic mechanisms of these traits in YZD goats.

## Figures and Tables

**Table 1:** Categorisation information of four traits in YZD goats.

Trait/Number	Samples/Number	
	Trait 1	Trait 0
Litter size/154	More than 2 lambs/7	1 to 2 lambs/147
Coat colour/196	Brown/10	White/186
Black middorsal stripe ^1^/196	Present/77	Absent/119
Skin colour/205	Pigmented/66	Unpigmented/139

^1^ The black middorsal stripe is a special coat pattern phenotype characterised by a 3–5-cm-wide black stripe of back of the body along the spine.

**Table 2:** Annotation of genome-wide significant SNPs and annotated genes for four traits in the YZD goat breed.

Trait	CHR ^1^	SNP Marker	Position	*p* Value	Annotated Gene
Litter size	11	snp54094-scaffold824-899720	57366312	6.87 × 10^−7^	Near to *LRRTM4* (leucine rich repeat transmembrane neuronal 4)
Coat colour	12	snp55048-scaffold842-324525	69164369	1.53 × 10^−7^	*LOC102187779*
	26	snp11508-scaffold142-1990450	23574535	6.90 × 10^−8^	*SORCS3* (sortilin-related VPS10 domain-containing receptor 3)
Black dorsal stripe	18	snp56013-scaffold873-22716	14190132	2.43 × 10^−9^	*TCF25* (transcription factor 25)

^1^ Chr, Chromosome.

**Table 3:** Descriptive statistics of runs of homozygosity (ROH) number and length by ROH length class.

Class	ROH Number	Percent (%)	Mean Length	Genome Coverage (%)
ROH 2–4 Mb	2882	64.82	2.59	0.30
ROH 4–8 Mb	756	17.00	5.45	0.16
ROH 8–16 Mb	426	9.58	10.84	0.18
>16 Mb	382	8.59	27.92	0.42
Total	4446	100	6.04	1.06

## Data Availability

The supplementary data can be accessed at https://figshare.com/s/7290686bce4994946cd7 (accessed on 25 February 2023). DOI: 10.6084/m9.figshare.20723674.

## Supplementary Materials

The following supporting information can be downloaded at: https://www.mdpi.com/article/10.3390/genes14061183/s1, Figure S1: The principal component analysis (PCA) result of 206 YZD goats. Figure S2: Q-Q plots for litter size and three morphological traits in YZD goats. Table S1: The GWAS phenotypic data. Table S2: The GWAS genotypic data. Tables S3–S6: The GWAS result for litter size, coat colour, black middorsal stripe and skin colour, respectively. Table S7: The SNPs obtained with estimated |iHS| scores by selection signature analyses. Table S8: A summary of genes from iHS in YZD goats. Table S9: GO and KEGG enrichment analysis of selected genes by iHS in YZD goats. Table S10: ROH result. File S1: Supplementary file 1.
