# Supplementary material for: Identifying Candidate Genes for Litter Size and Three Morphological Traits in Youzhou Dark Goats Based on Genome-Wide SNP Markers
Source: Genes (Basel). 2023 May 29;14(6):1183. doi: 10.3390/genes14061183 (PMC10298679; doi:10.3390/genes14061183)
Supplement: Supplementary file 1 [file genes-14-01183-s001.zip › Supplementary file 1.pptx]

## Slide 1
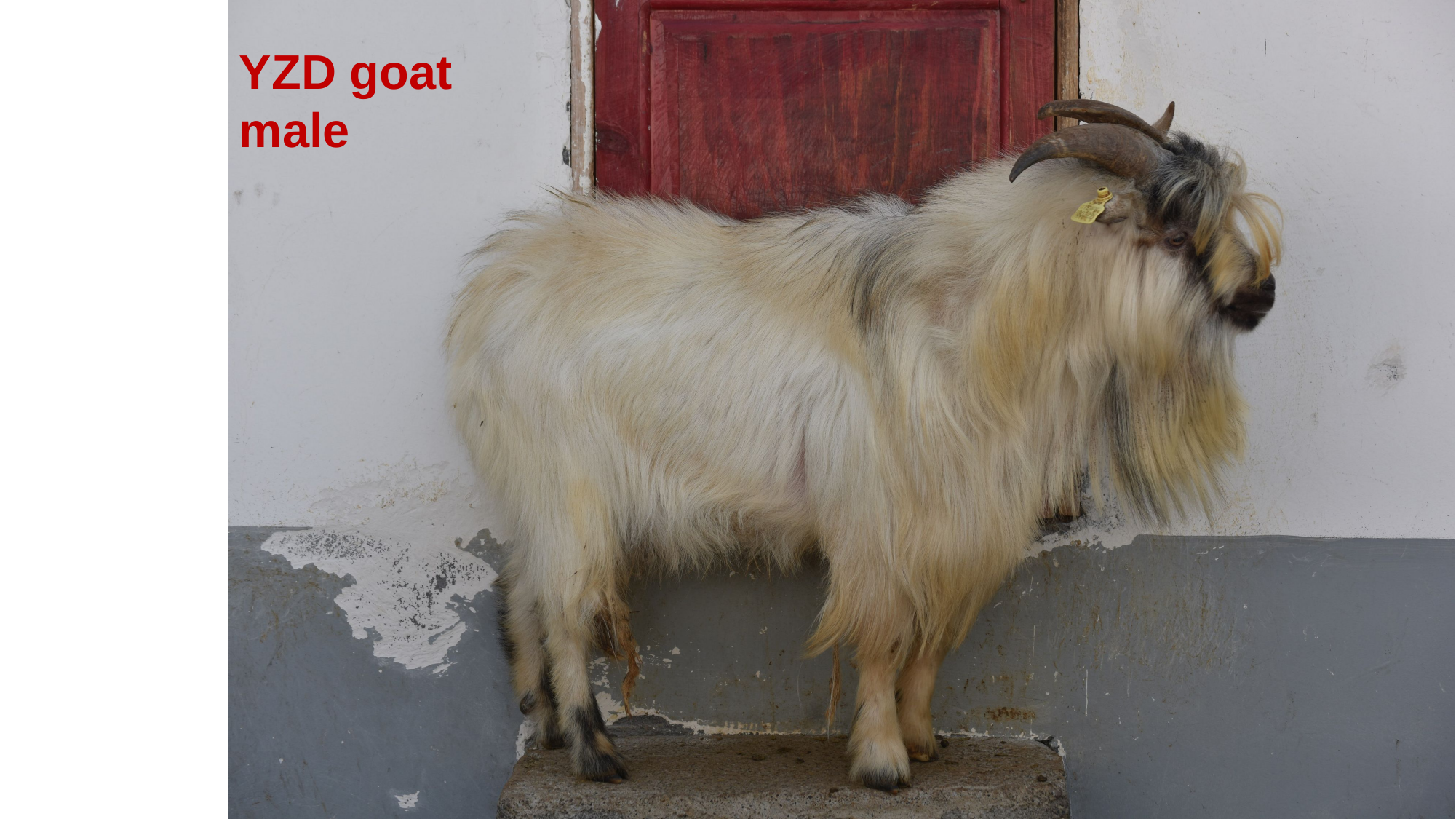

YZD goat male

## Slide 2
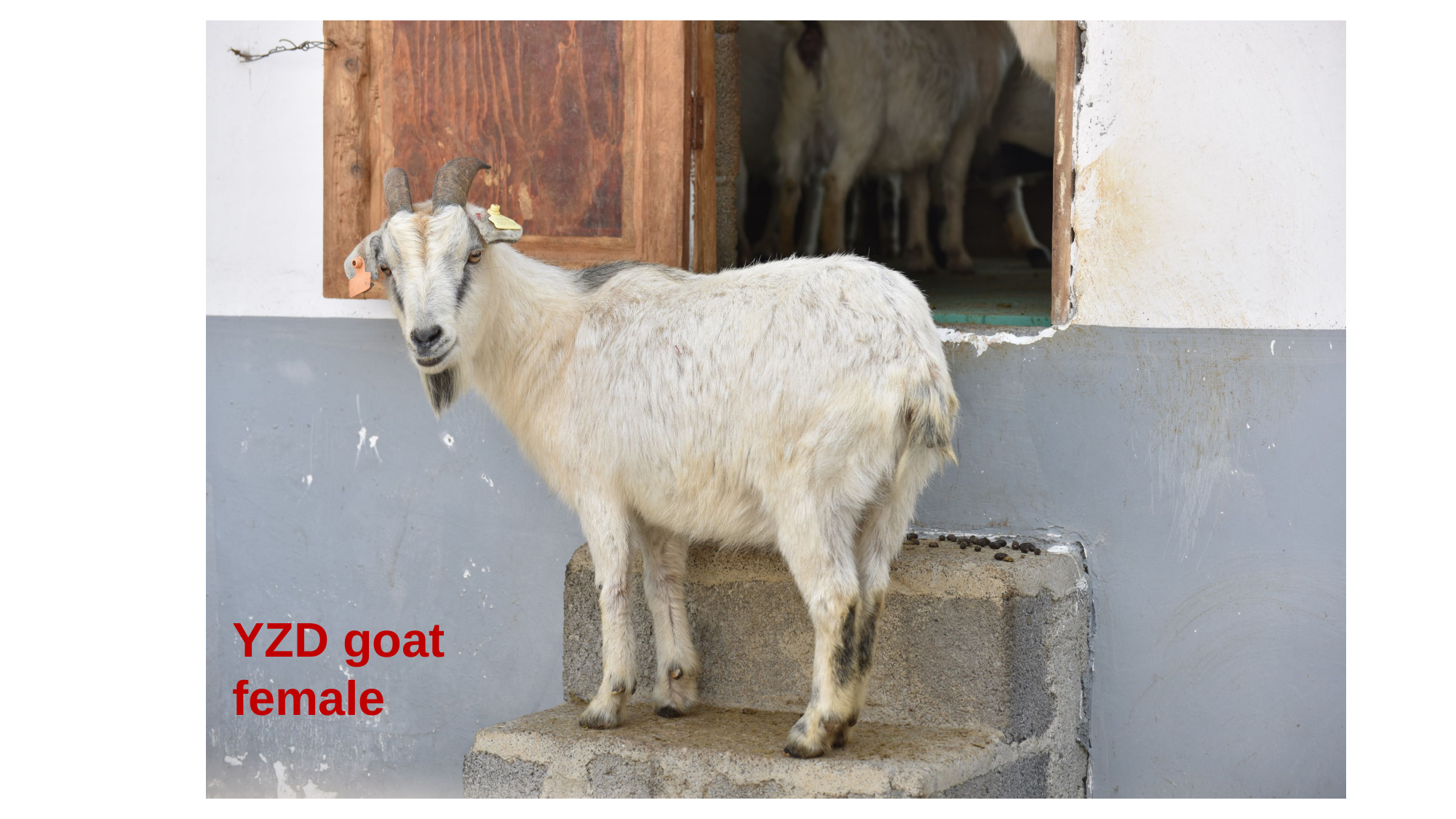

YZD goat female

## Slide 3
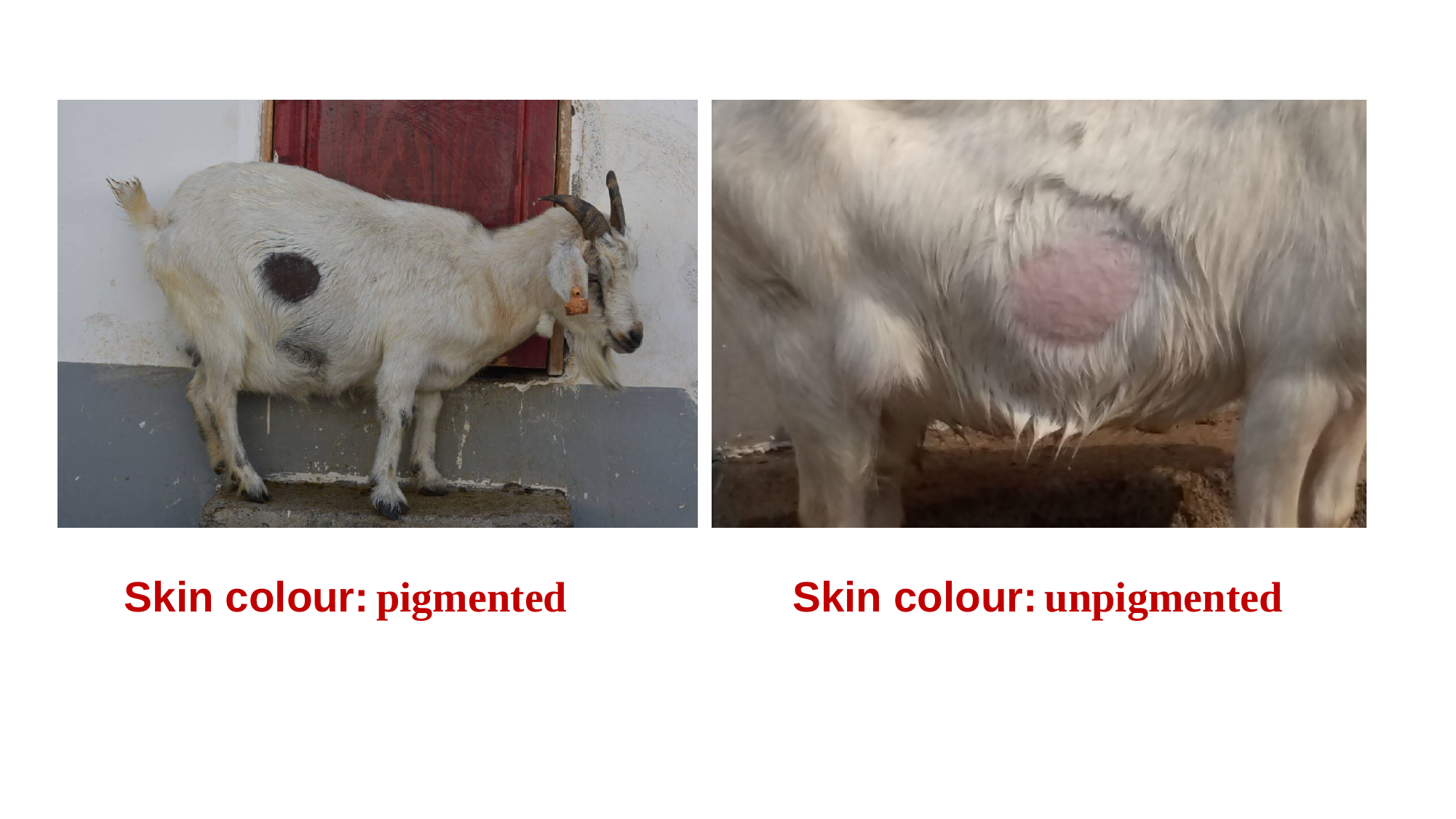

Skin colour: pigmented
Skin colour: unpigmented

## Slide 4
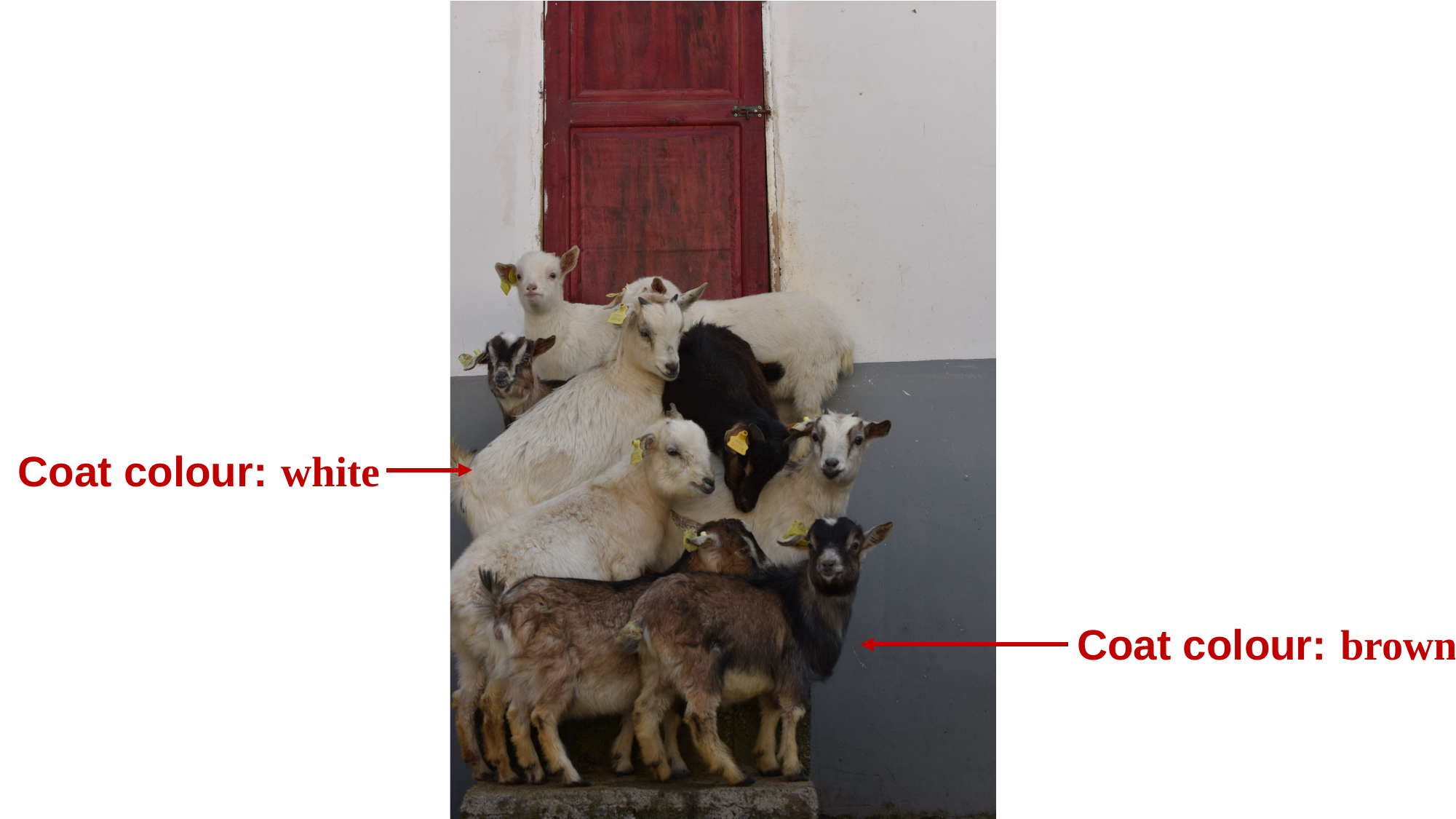

Coat colour: white
Coat colour: brown

## Slide 5
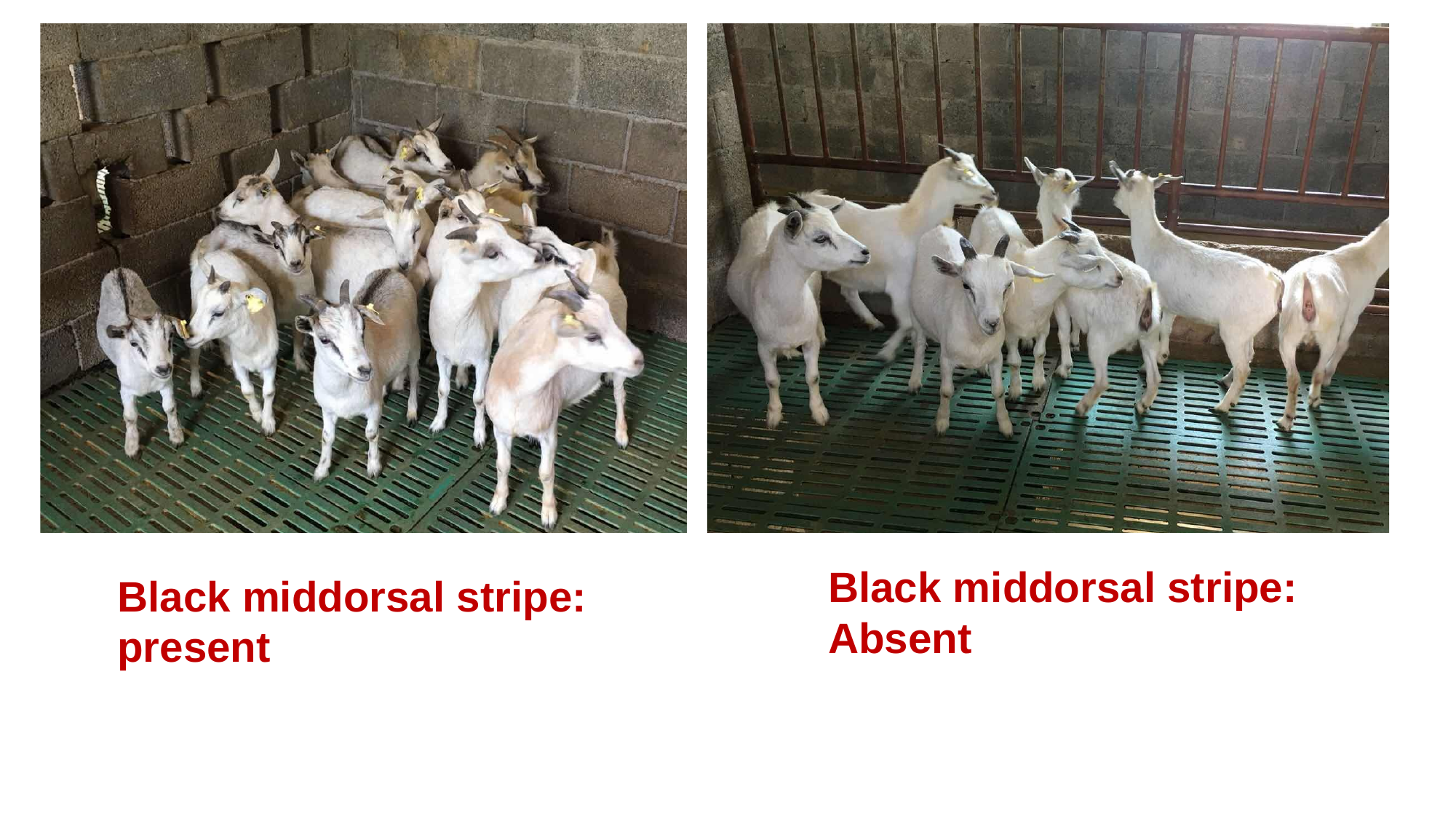

Black middorsal stripe: Absent
Black middorsal stripe: present

## Slide 6
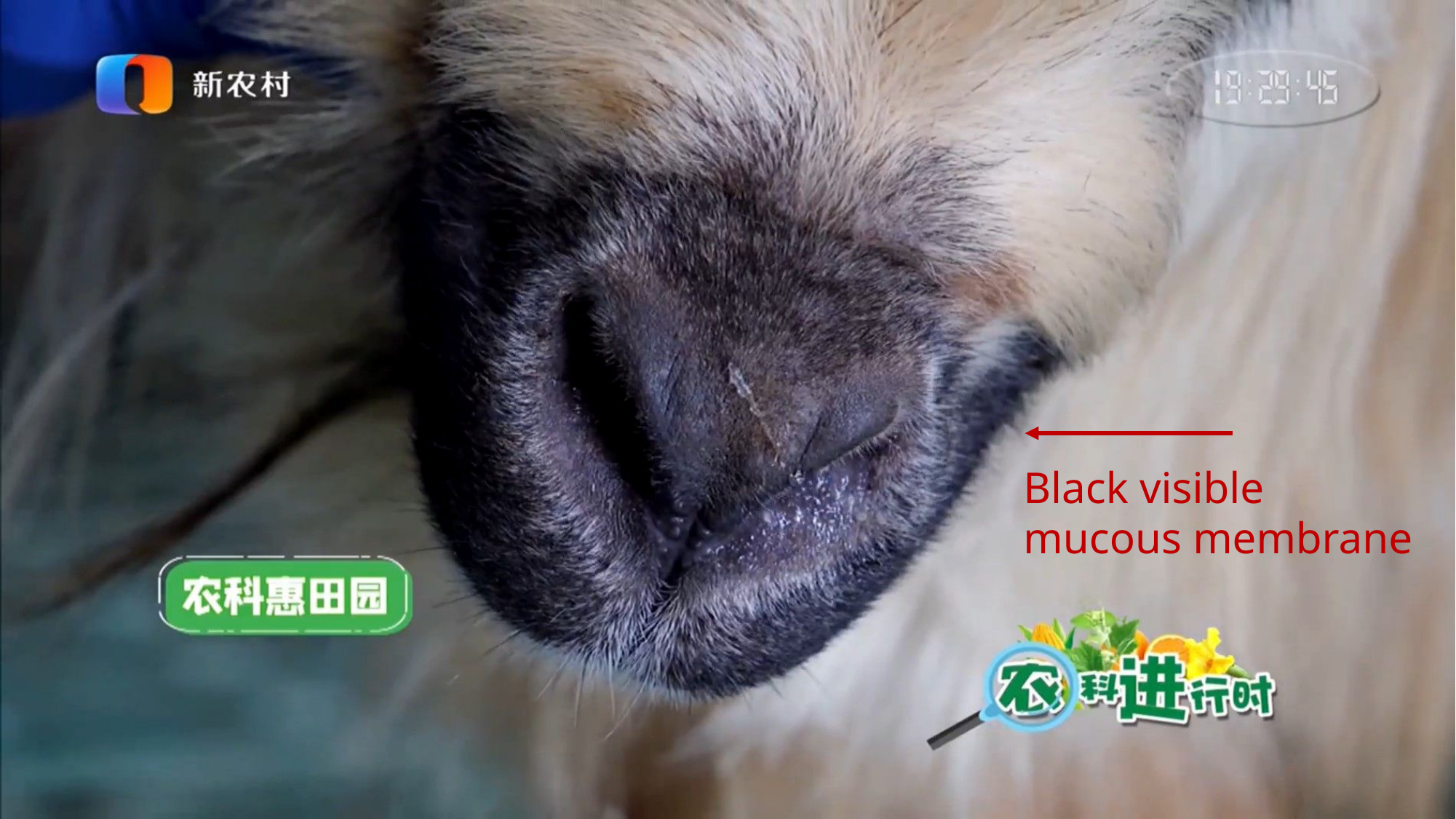

Black visible
mucous membrane

## Slide 7
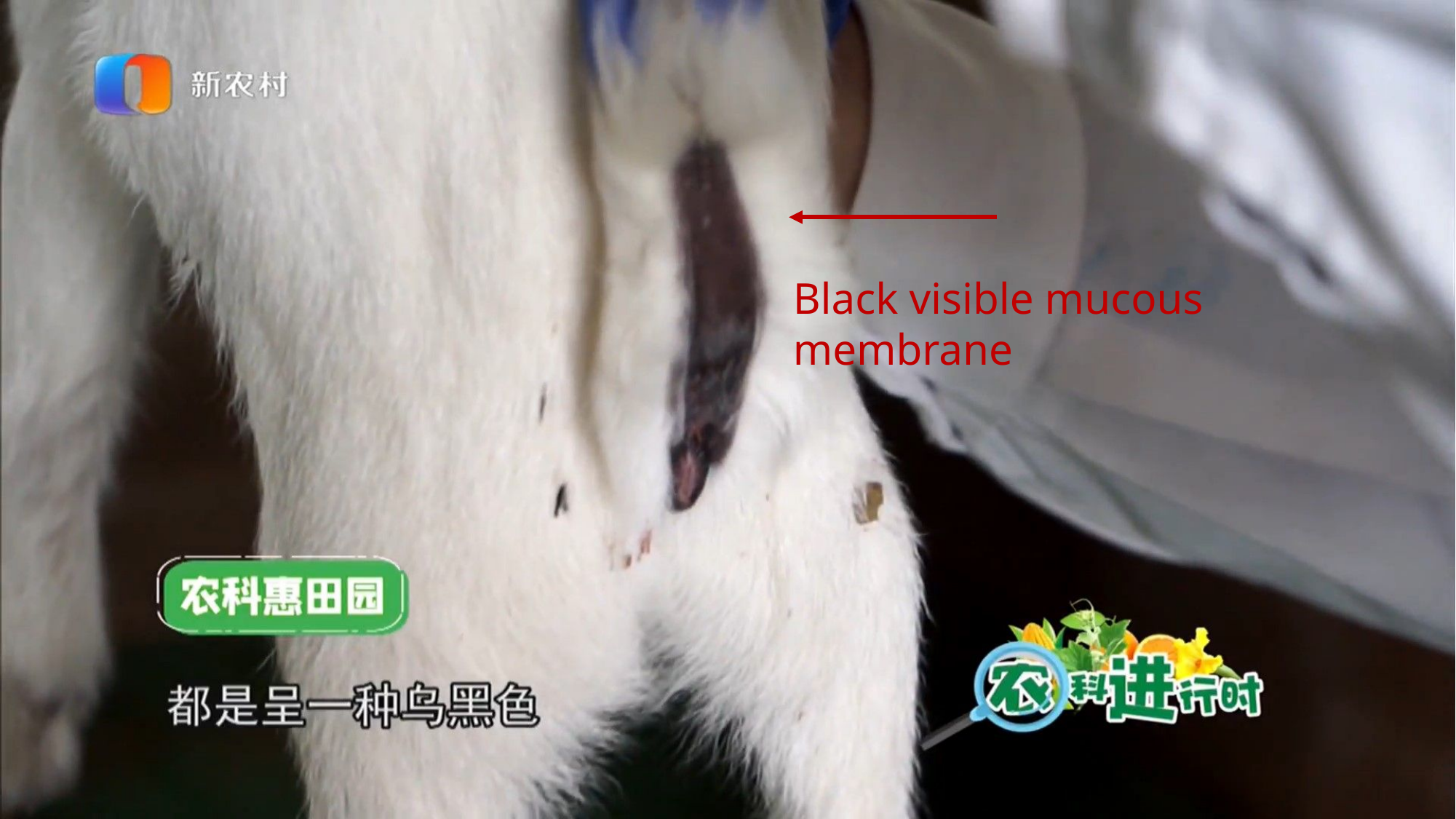

Black visible mucous membrane

## Slide 8
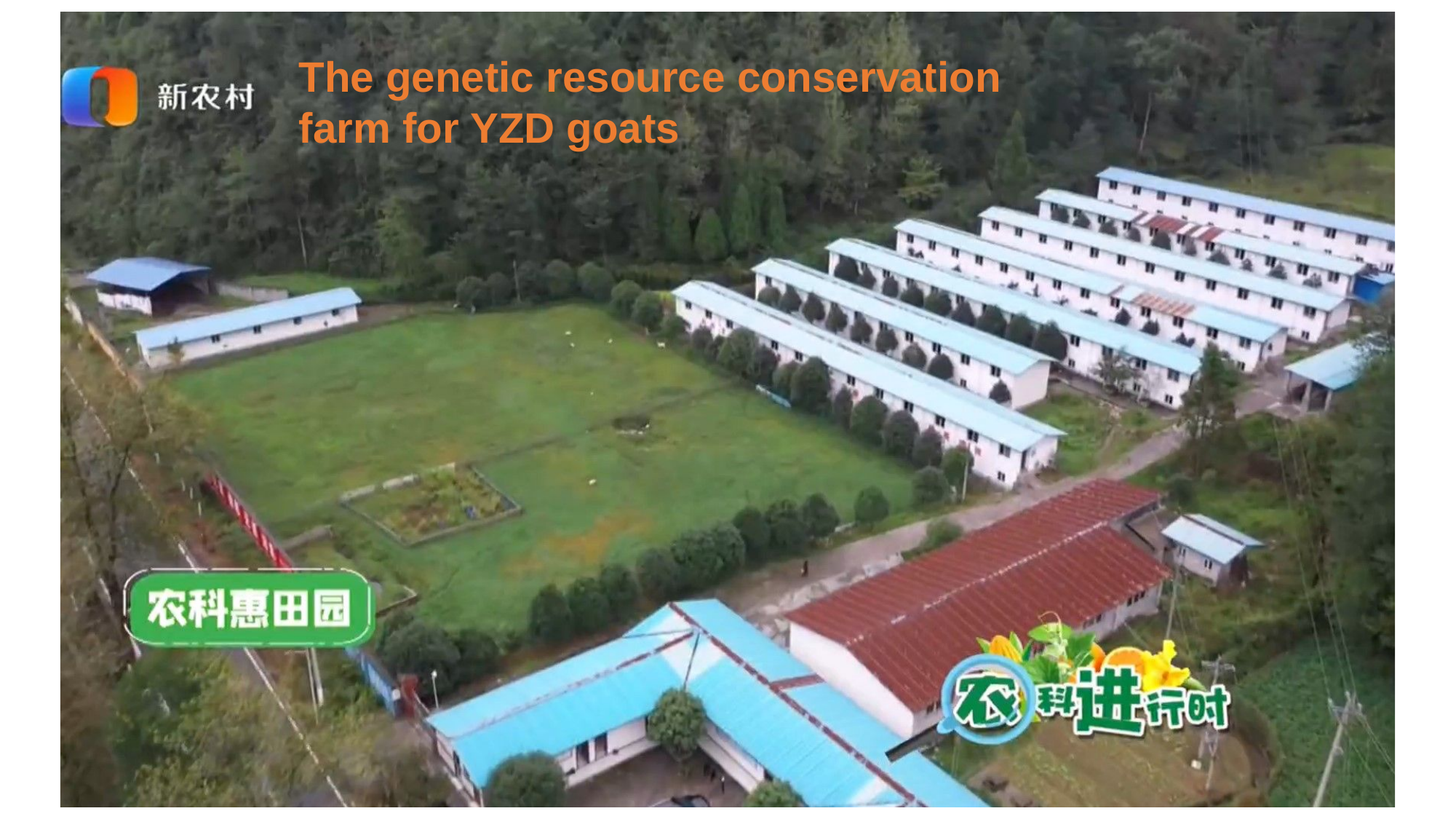

The genetic resource conservation farm for YZD goats
