# Supplementary figures and images for: Identifying Candidate Genes for Litter Size and Three Morphological Traits in Youzhou Dark Goats Based on Genome-Wide SNP Markers
Source: Genes (Basel). 2023 May 29;14(6):1183. doi: 10.3390/genes14061183 (PMC10298679; doi:10.3390/genes14061183)

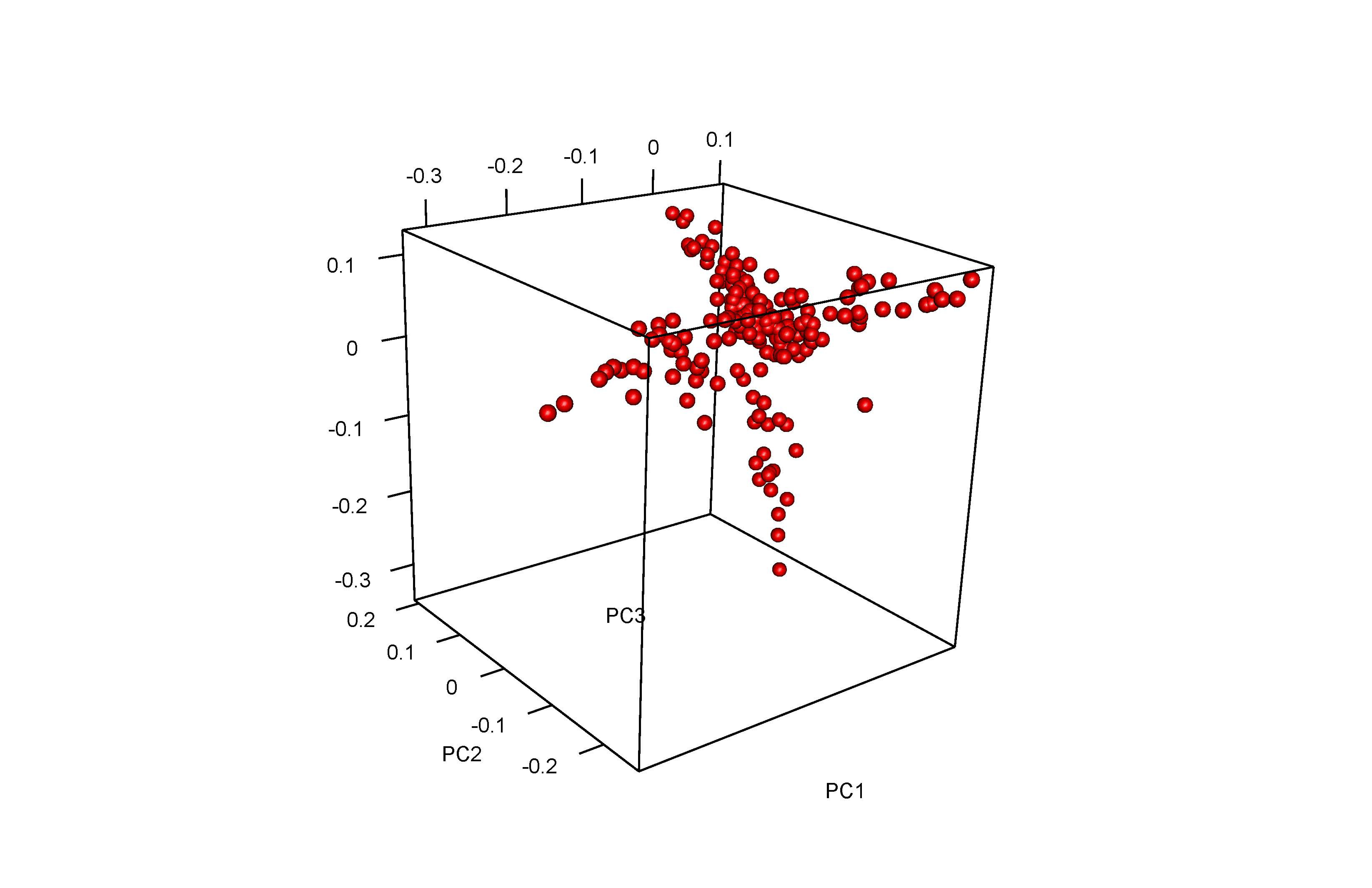

Supplement: Supplementary file 1 [file genes-14-01183-s001.zip › Figure S1.tif]

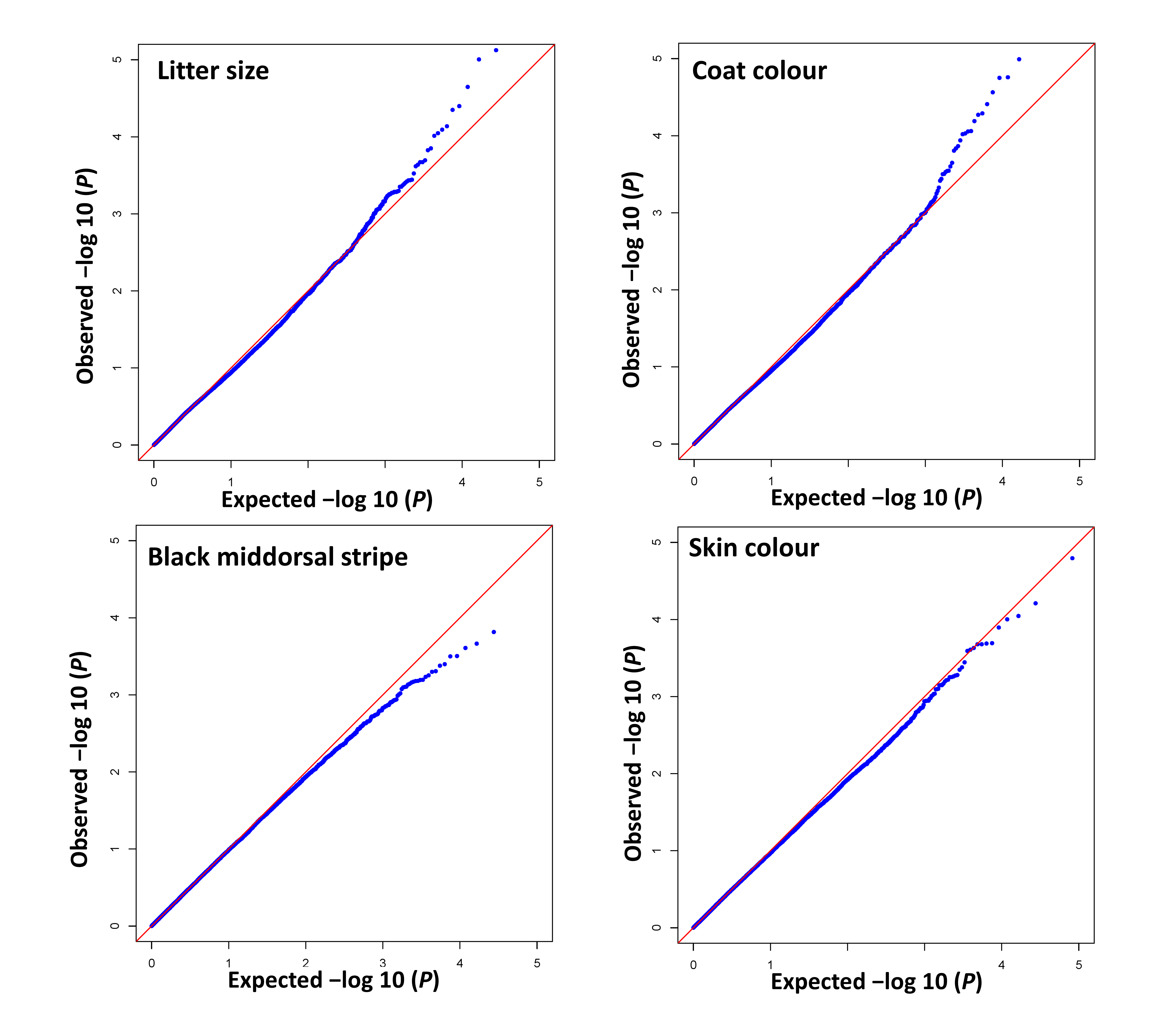

Supplement: Supplementary file 1 [file genes-14-01183-s001.zip › Figure S2.tif]
